# Effects of Rehabilitation Robot Training on Physical Function, Functional Recovery, and Daily Living Activities in Patients with Sub-Acute Stroke

**DOI:** 10.3390/medicina60050811

**Published:** 2024-05-15

**Authors:** Se-Young Kim, Mi-Young Lee, Byoung-Hee Lee

**Affiliations:** 1 Graduate School of Physical Therapy, Sahmyook University, Seoul 01795, Republic of Korea; seyoungk02@naver.com; 2 Department of Physical Therapy, Sahmyook University, Seoul 01795, Republic of Korea; mylee@syu.ac.kr

**Keywords:** sub-acute stroke, robot rehabilitation, muscle strength, balance, functional status, activities of daily living

## Abstract

Stroke often results in sensory deficits, muscular weakness, and diminished postural control, thereby restricting mobility and functional capabilities. It is important to promote neuroplasticity by implementing task-oriented exercises that induce changes in patients. Therefore, this study aimed to investigate the effects of rehabilitation robot training on physical function, functional recovery, and activities of daily living (ADLs) in patients with subacute stroke. The study participants were patients with subacute stroke receiving treatment at Hospitals A and B. They were selected as research subjects based on selection and exclusion criteria. The experimental group received rehabilitation robot training in sessions of 30 min, five times weekly, for a total of 20 sessions over four weeks. Conversely, the control group underwent standard rehabilitation equipment training with an identical frequency, duration, and number of sessions. Measurements were taken before and after the training period to assess changes in physical function, functional recovery, and activities of daily living using tools such as the MMT, BBS, FBG, FAC, FIM, and MBI. The results were as follows: in the within-group comparison, the rehabilitation robot training group showed significant differences in MMT, BBS, FBG, FAC, FIM, and MBI (*p* < 0.05), while the control group showed significant differences in FIM (*p* < 0.05). Statistically significant differences were observed in the time, group, and time × group interaction effects among the MMT, static seated FBG, dynamic seated FBG, FIM, and MBI (*p* < 0.05). Based on these results, rehabilitation robotic training resulted in significant improvements in physical function, functional recovery, and activities of daily living in patients with subacute stroke. Based on these findings, providing a basic protocol for a rehabilitation program that applies rehabilitation robot training to patients with subacute stroke may offer more effective treatment and outcomes in the future.

## 1. Introduction

Stroke is the leading cause of disability worldwide and the third most common cause of death [1]. The mortality rate from cerebrovascular diseases in South Korea has increased from 42.6 deaths per 100,000 people in 2020 to 44.0 deaths in 2021. Additionally, the number of individuals who will receive emergency room treatment for stroke in South Korea in 2021 will be 120,584, which represents an approximately 29.7% increase compared with 93,670 in 2014 [2]. Stroke is a serious cerebrovascular disease that affects either the local or global brain [3]. Stroke occurs when a part of the brain is deprived of the necessary blood flow due to either a sudden interruption of blood supply to a part of the brain or a rupture of blood vessels in the brain, leading to blood invading the surrounding areas [4]. Neurological symptoms of stroke affect cognitive domains, including attention, memory, language, and orientation [5]. It manifests as loss or restriction of muscle function leading to limitation or loss of movement, mobility, and functional abilities [6]. Additionally, it involves sensory impairment, changes in muscle tone and reflexes, postural control and balance disorders, executive dysfunction, unilateral neglect, and sensory deficits [7].

Interventions for stroke rehabilitation follow three basic principles: adaptation, regeneration, and neuroplasticity. It aims to promote patient independence and restore functional impairments [8]. Neuroplasticity is defined as the ability of the nervous system to reorganize its structure, function, and connections in response to internal and external stimuli [9]. The rehabilitation of stroke patients aims to promote neuroplasticity through exercise-based therapies such as constraint-induced movement therapy (CIMT) [10] and task-oriented exercises [10], as well as robot-assisted rehabilitation [11], functional electrical stimulation [12], core stabilization exercises [13], MOTOmed training [14], task-specific circuit training [15], and cognitive and motor dual-task gait training [16]. Therefore, for stroke recovery, high-dose concentrated training and repetitive practice of specific functional tasks are crucial [17].

Robot-assisted rehabilitation is known not only to enhance neuroplasticity, which plays a crucial role in motor control recovery in patients with stroke [18], but also to boost motivation, self-efficacy, and determination [19]. The use of robotic devices provides advantages such as frequency/intensity modulation of stimuli, support for voluntary action intention, and enrichment of sensory–motor information to enhance interaction. These principles support experience-dependent neuroplasticity that underlies rehabilitation-induced recovery [20]. Electric machines/robotic devices that automate lower limb exercises have been developed to enhance the safety, intensity, and standardization of training, thus assisting the role of physical therapists. These devices are designed to generate complex multisensory stimuli and provide extensive external biofeedback to patients [21]. Rehabilitation robots utilize neuroplasticity to induce changes in patients [22]. The rehabilitation robot Erigo^®^Pro (Hocoma AG, Zurich, Switzerland, 2014) is a robot tilt table (RTT) that provides gradual verticalization and weight-bearing. It includes a lower limb stepping device for periodic leg exercises and incorporates functional electrical stimulation (FES), preventing orthostatic hypotension during verticalization and ensuring stability in an upright position [23,24].

Functional electrical stimulation (FES) therapy is designed to stimulate paralyzed muscles through electrical stimulation of nerve supplies. It has been reported to improve spasticity control and gait speed in both acute and chronic stroke patients [25]. Erigo^®^Pro provides a safe rehabilitation strategy not only for restoring motor and cognitive functions but also for enhancing neuroplasticity within the sensory–motor and vestibular systems, thereby potentially preventing chronic conditions [18].

Current research on neurodevelopmental treatment (NDT) in stroke patients has demonstrated many effects on physical function, functional recovery, and activities of daily living [26,27,28]. However, studies on the effects of rehabilitation robotic training on physical function, functional recovery, and activities of daily living in patients with acute stroke are lacking.

Therefore, in this study, we aimed to compare and analyze physical function, functional recovery, and activities of daily living between a rehabilitation robot training group and a control group of patients with acute stroke. This is not only to provide fundamental data necessary for rehabilitation robot training programs targeting acute stroke patients in the future, but also to utilize it as useful information for therapists, stroke patients, and caregivers in evaluating and designing treatment processes.

## 2. Materials and Methods

### 2.1. Subjects

In this study, we targeted 35 adults diagnosed with stroke within 6 months of receiving treatment at Hospitals A and B in Seoul and Gyeonggi Province. Before recruiting participants for this study, we performed a power analysis using G*Power version 3.1.9.7 (Heinrich-Heine Universität, Düsseldorf, Germany); additionally, repeated measures ANOVA was used to assess within–between interaction. An effect size f of 0.25 was obtained for all the outcome measures, with a α error probability of 0.05, to minimize the type 1-β error probability of 95%. The number of groups was two and the number of measurements was five. As the estimated target sample size was 32, we recruited 45 participants who underwent physical therapy.

The inclusion criteria were as follows: individuals who were able to walk normally before the onset of stroke, individuals with leg lengths between 75 and 100 cm, individuals weighing less than 135 kg, and individuals who had not participated in any other rehabilitation robotic training program within the past 3 months. Individuals with contraindications to lower limb weight-bearing, such as fractures, skin damage, pressure ulcers, uncontrolled hypertension or orthostatic hypotension, cardiovascular diseases or heart failure, malignant diseases, pulmonary diseases, neurological disorders, or other underlying conditions that would make them unable to tolerate robot-assisted gait therapy, were excluded.

All participants signed a consent form after the procedure, and the purpose of the study was explained. This study was approved by the Institutional Review Board of Sahmyook University (approval number: SYU 2023-03-011-001). The trial protocol was registered retrospectively with the Clinical Research Information Service of the Republic of Korea (KCT0008528). Participants fully understood the objectives and procedures used in the study. The study adhered to the ethical principles of the Declaration of Helsinki.

### 2.2. Experimental Procedure

The general characteristics of the 40 eligible participants who met the selection criteria were assessed before the experiment. Sex, age, weight, and height were confirmed through questions and measurements with the participants and their guardians. Participants in both groups underwent pretesting and were randomly divided into two groups using the Research Randomizer program (http://www.randomizer.org/, accessed on 10 April 2024) to minimize errors related to the experiment. They were then assigned to the therapists to minimize bias and conduct the experiment.

This study aimed to investigate the effects of rehabilitation robot training in patients with acute stroke. The participants were divided into two groups: an experimental group that received rehabilitation robot training and a control group that received conventional rehabilitation equipment training. Physical function, functional recovery, and activities of daily living were evaluated and compared between groups. The rehabilitation robot training group underwent training sessions with the rehabilitation robot Erigo^®^Pro (Erigo^®^Pro; Hocoma AG, Zurich, Switzerland, 2014) and the rehabilitation equipment MOTOmed viva2 light (MOTOmed viva2 light; RECK-Technik GmbH & Co. KG, Betzenweiler, Germany, 2012). Each training session occurred five times a week for 30 min per session, totaling 20 sessions over four weeks. The control group received training with rehabilitation equipment only, and the training was conducted five times a week for 30 min per session, totaling 20 sessions over four weeks. To measure changes in participants before and after training, physical function was assessed using MMT; functional recovery was assessed using BBS, FBG, and FAC; and activities of daily living were assessed using FIM and MBI. The difference between the scores before and after training was calculated to determine the change in scores for each period. The degree of improvement in each period was compared between the two groups.

### 2.3. Training Program

Patients with acute stroke were divided into rehabilitation robot training and control groups. They participated in the program according to a prescheduled itinerary. Two research assistants were assigned for educational and evaluative purposes, and the research was conducted accordingly. Before conducting each evaluation, explanations were provided regarding the assessment methods, and training programs were explained to minimize errors that could occur during the experiment. Additionally, this study included physical, occupational, and speech therapists who had acquired specialized knowledge and completed professional education courses. Both groups received physical therapy, occupational therapy, and speech therapy for 30 min each, five times a week, for a total of 20 sessions over four weeks.

#### 2.3.1. Rehabilitation Robot Training

Erigo^®^ Pro is designed as a training tool for lower limb stepping mechanisms, including a robot tilt-table (RTT), and it simultaneously applies functional electrical stimulation (FES) to enhance muscle activity further. For the rehabilitation robot training process using Erigo^®^ Pro in this study, the participants wore shoes and laid flat on the inclined platform. They adjusted their hip joints to the mat and harness lines and aligned their feet on the footrest. Their shoulders, waist, pelvis, and ankles were secured and fixed with belts and straps. The knee cuff was adjusted to a position just above the three fingers (approximately 3–4 cm) above the kneecap, ensuring the safety of the subject during the movement of the hip and knee joints.

For a progressive training protocol, the adjustable parameters include tilt angle, loading, and cadence. The difficulty level was set by adjusting these parameters based on the subject’s cardiovascular condition, fatigue, concentration, adaptability, and other outcomes. The amount of passive assistance could be adjusted separately for each side (left and right) based on the condition of the subject.

The ranges of motion (ROM) of the hip and knee joints of each leg were measured using sensors integrated into a training device. The training device software controlled the robotic leg movement within the ROM limits. The tilt angle started at 20° and increased to 40°, 60°, and 80° sequentially, while the cadence (steps per minute) gradually increased from 40° to 50°. The rehabilitation robot training lasted for 30 min/day, five days a week for four weeks, totaling 20 sessions. The time spent wearing the device and adjusting the computer was not included in the training time. The rehabilitation robot training group also underwent rehabilitation device training for 30 min per day, five days a week for four weeks, for a total of 20 sessions (Table 1).

#### 2.3.2. Rehabilitation Device

The rehabilitation device used for rehabilitation device training was the MOTOmed viva2 light. The training process was as follows: although the MOTOmed viva2 light allows for exercise of both the upper and lower limbs, only lower limb exercises were conducted. The subjects sat in a wheelchair or fixed chair and placed their feet on the footrest, assuming proper foot alignment of their feet. Their feet, calves, and thighs were secured and fixed with attached straps.

The MOTOmed viva2 light can be set to either passive exercise, driven by the motor, or active exercise, using an individual’s own strength. During passive exercise, speed control is possible within the range of 0–60 revolutions per minute (rpm), gradually increasing from 40 to 50 rpm. During active exercise, resistance can be adjusted to a maximum of 20 levels, gradually increasing from level 1 to level 4. The difficulty level was set by adjusting parameters such as the tilt angle, cadence, speed, and resistance based on the subject’s cardiovascular condition, fatigue, concentration, adaptability, and other outcomes. The rehabilitation device training lasted for 30 min/day, five days a week, for four weeks, totaling 20 sessions. The time allocated for wearing the device and adjusting the equipment was not included in the training time.

#### 2.3.3. Outcome Measures

Functional recovery was assessed using the Berg Balance Scale (BBS), Functional Ambulation Category (FAC), and Functional Balance Grade (FBG). Daily living activities were measured using the Functional Independence Measure (FIM) and the Modified Barthel Index (MBI).

In this study, physical function was assessed using manual muscle testing (MMT) originally devised by Wright and Lovett. The muscle strengths of the hip joint, knee joint, dorsiflexors, and plantar flexors of the ankle joint were assessed. The evaluation scored the ability to contract muscles or muscle groups against resistance or gravity, enabling a quantitative evaluation. 

The scoring system used was as follows: Zero (0 points), Trace (1 point), Poor- (2- points), Poor (2 points), Poor+ (2+ points), Fair- (3- points), Fair (3 points), Fair+ (3+ points), Good (4 points), and Normal (5 points). This measurement tool demonstrates high reliability, with intra-rater and inter-rater reliability coefficients (ICC) both exceeding 0.9 [29].

In this study, functional recovery included both balance and walking abilities. Balance ability was measured using Berg’s Balance Scale (BBS) and the Functional Balance Grade (FBG), assessing sitting and standing positions and static and dynamic balance abilities. Functional walking ability was evaluated using the Functional Ambulation Category (FAC) criteria. Balance was assessed quantitatively through direct observation using the Berg Balance Scale (BBS), which evaluates balance and the risk of falls. The scale consists of three areas and 14 items. Each item is scored on a five-point ordinal scale ranging from 0 to 4, with a total score of 56 points. The three areas are sitting (one item), standing (eight items), and postural changes (five items). Scores below 45 of 56 points were classified as being at risk of falls. This measurement tool demonstrates high reliability when used with stroke patients, with inter-rater reliability coefficients (ICCs) ranging from 0.92 to 0.98, an intra-rater reliability coefficient (CC) of 0.97, and a test–retest reliability coefficient (ICC) of 0.98 [30].

In this study, balance was assessed using the Functional Balance Grade (FBG), which evaluates the subject’s sitting and standing positions, as well as static and dynamic balance abilities. This scale allows for the classification of functional balance levels from Poor to Normal, distinguishing between static and dynamic balance abilities in the sitting and standing positions. Progression from Poor to Normal indicates an improvement in functional balance, with Normal representing the highest level of balance function. The measurement tool demonstrates excellent intra-rater reliability, with intra-class correlation coefficients (ICCs) ranging from 0.93 to 0.96 [31].

Walking was assessed using the Functional Ambulation Category (FAC). The Functional Ambulation Category (FAC) is an efficient assessment method that has been validated for its reliability and validity in quickly and easily evaluating the level of assistance required for walking and the level of independence in stroke patients [32]. The FAC utilizes a six-point scale ranging from 0 to 5, where 0 represents an inability to walk and 5 represents independent walking. Higher scores indicated better mobility and walking ability. This measurement tool demonstrated high reliability, with a test–retest reliability of Cohen’s kappa = 0.95 and an inter-rater reliability of kappa = 0.905 [32].

In this study, the subjects’ performance in activities of daily living was objectively evaluated using the Functional Independence Measure (FIM). The FIM consists of 18 items categorized into six domains: self-care, sphincter control, mobility, locomotion, communication, and social cognition. Each item is scored on a scale of 1 to 7 based on the level of dependence, with a total score of 126 points. This measurement tool demonstrates high reliability, with an inter-rater reliability coefficient of r = 0.83 [33].

In this study, independent functioning and performance of activities of daily living were measured using the Modified Barthel Index (MBI). The MBI is an assessment tool for activities of daily living that was revised and supplemented by Shah et al. in 1984 based on the Barthel Index developed by Barthel et al. It consists of ten items and utilizes a five-point scoring system based on the level of assistance provided. The total score is 100 points, and the scores for each item related to daily activities are summed to record the score. The higher the score, the more independent the performance of activities of daily living.

This measurement tool demonstrates excellent reliability, with an inter-rater reliability coefficient (ICC) of 0.99 and an intra-rater reliability coefficient (ICC) of 0.99 [34].

#### 2.3.4. Data Analysis

All statistical analyses were performed using the SPSS ver. 22.00 software (SPSS Inc., Chicago, IL, USA). To describe the general characteristics of the participants, we calculated their means and standard deviations. Additionally, we used the Shapiro–Wilk test to assess the normal distribution of the data. We used the chi-square test to determine homogeneity between the two groups before the intervention. We used a paired *t*-test to analyze within-group pre–post differences in BBS, FIM, and MBI. For MMT, FBG, and FAC, we used the Wilcoxon signed-rank test to assess the within-group pre–post differences. We used repeated measures ANOVA to evaluate the interaction between the groups and time. Statistical significance was set at a *p*-value < 0.05.

## 3. Results

### 3.1. General Characteristics of Participants

This study aimed to investigate the effects of rehabilitation robot training in patients with subacute stroke. The participants were divided into rehabilitation robot training and control groups. There were no significant differences between the two groups in terms of sex, age, height, weight, cause of onset, lesion site, or onset period, indicating homogeneity (Table 2).

### 3.2. Comparison of Physical Function

The pre- and post-intervention changes in manual muscle testing (MMT) for the rehabilitation robot training group revealed statistically significant increases in the hip flexors from 2.40 to 4.60 points. Additionally, the hip extensors, knee flexors, knee extensors, dorsiflexors, and plantar flexors of the ankle joint showed statistically significant increases after the intervention (*p* < 0.05). In the control group, there were no significant changes in the hip extensors pre- or post-intervention. However, the hip flexors, knee flexors, knee extensors, dorsiflexors, and plantar flexors showed increases in MMT scores after the intervention, although these changes were not statistically significant. Upon comparison between groups, there were statistically significant differences in MMT scores for hip flexors, hip extensors, knee flexors, knee extensors, dorsiflexors, and plantar flexors. These differences were observed in the main effects of the time and group and the interaction effect between the time and group (*p* < 0.05) (Table 3).

### 3.3. Comparison of Functional Recovery

In the rehabilitation robot training group, the Berg Balance Scale (BBS) increased significantly from 1.60 to 6.00 points after the intervention (*p* < 0.05). In contrast, in the control group, the BBS increased from 0.26 to 0.67 points after the intervention, but this difference was not statistically significant. No significant differences were observed between the groups; however, there was a significant interaction effect between the time and group (*p* < 0.05).

In the rehabilitation robot training group, there were significant increases in the static seated posture FBG from 0.65 to 1.50 points, dynamic seated posture FBG from 0.35 to 1.00 points, and static standing posture FBG from 0.15 to 0.55 points after the intervention (*p* < 0.05). However, there was no significant difference observed in the dynamic standing posture FBG, which increased from 0.10 to 0.35 points (*p* = 0.056). In the control group, there was an increase in the static seated posture FBG from 0.20 to 0.40 points after the intervention; however, this difference was not statistically significant.

Between-group comparisons revealed statistically significant differences in static seated posture FBG and dynamic seated posture FBG levels in terms of the main effects of the time and group and the interaction effect between the time and group (*p* < 0.05).

In the rehabilitation robot training group, there was a significant increase in the Functional Ambulation Category (FAC) from 0.50 to 0.65 points after the intervention (*p* < 0.05). In the control group, there was an increase in the Functional Ambulation Category (FAC) from 0.00 to 0.07 points after the intervention, but this difference was not statistically significant. (Table 4).

### 3.4. Comparison of ADL

In the rehabilitation robot training group, there was a significant increase in the Functional Independence Measure (FIM) from 23.25 to 36.30 points after the intervention (*p* < 0.05). Additionally, the Modified Barthel Index (MBI) significantly increased from 4.15 to 16.45 points after the intervention (*p* < 0.05). 

In the control group, there was a significant increase in the Functional Independence Measure (FIM) from 20.07 to 22.40 points after the intervention (*p* < 0.05). There was an increase in the Modified Barthel Index (MBI) from 0.73 to 2.47 points after the intervention, but the difference was not statistically significant.

Upon comparison between groups, both the FIM and MBI showed statistically significant differences in the main effects of the time, group, and the interaction effect between the time and group (*p* < 0.05) (Table 5).

## 4. Discussion

### 4.1. Physical Function

In terms of physical function, not only does hemiparesis affect muscle strength on the side affected by stroke, it also reduces muscle strength on the opposite side, which closely influences functional performance [35]. For stroke patients, these physical functions are important factors, and robot-based rehabilitation that promotes neuroplasticity can induce changes in lower limb muscle strength [36]. This study compared the changes in lower limb muscle strength in a rehabilitation robot training group in terms of physical function. The results showed that flexion and extension of the hip, knee, and ankle joints significantly increased (*p* < 0.05) based on manual muscle testing (MMT). There were significant differences in the flexion and extension of the hip, knee, and ankle joints based on manual muscle testing (MMT) between the rehabilitation robot training and control groups in the time × group interaction effect (*p* < 0.05). When examining previous studies on rehabilitation robot training and lower limb muscle strength in stroke patients, Kumar et al. [37] divided 110 acute stroke patients randomly into two groups: one receiving conventional physical therapy and the other undergoing ergo tilt table rehabilitation. Lower limb muscle strength was measured by manual muscle testing (MMT) before and after rehabilitation. 

Based on the measured values, they evaluated the improvement in outcome variables within each group and compared the groups. They performed manual muscle testing (MMT) based on movements at the hip, knee, and ankle joints and used the average score of these measurements as the overall lower limb muscle strength. In the conventional physical therapy group, there was a significant increase in MMT scores compared to baseline, starting at 1.25 points to 2.47 points after 30 days of treatment and increasing further to 3.36 points after 90 days (*p* < 0.001). In the ergo tilt table rehabilitation group, there was a significant increase in MMT scores compared to baseline: from 1.42 points to 2.88 points after 30 days of treatment and increasing further to 3.90 points after 90 days (*p* < 0.001). There was a significant difference in the MMT between the ergo tilt table rehabilitation group and the conventional physical therapy group 90 days after treatment (*p* < 0.05). There was a significant interaction effect of time × group on the MMT in all cases (*p* < 0.05). Kuznetsov et al. [38] divided 104 acute stroke patients into three groups: a robot tilt table group combined with functional electrical stimulation (ROBO–FES), a robot tilt table group without functional electrical stimulation (ROBO), and a tilt table group (control). They evaluated the improvement in outcome variables within each group and between the groups by measuring lower limb muscle strength using the Medical Research Council (MRC) muscle strength scale. In the ROBO–FES group, there was a significant increase in lower limb muscle strength from 2.10 points to 4.00 points. In the ROBO group, the strength increased significantly from 1.90 points to 3.40 points. Similarly, in the control group, there was a significant increase from 2.30 points to 3.40 points (*p* < 0.0001). In the ROBO–FES group, there was a significant difference in lower limb muscle strength compared to that in the control group (*p* < 0.05). There were no significant differences between the ROBO–FES and ROBO groups or between the ROBO and control groups in terms of lower limb muscle strength. The results of this study indicate, similar to the studies conducted by Kumar et al. [37] and Kuznetsov et al. [38], that robot-assisted rehabilitation training is an effective intervention method for increasing lower-limb muscle strength in subacute stroke patients, particularly in terms of flexion and extension of the hip, knee, and ankle joints. In this study, robot-assisted rehabilitation training involved automated movements of the hip joint, knee joint, and ankle joint, along with functional electrical stimulation (FES) embedded in the Erigo^®^Pro device. This combination facilitates muscle contraction in stroke patients with difficulty achieving active movement, potentially leading to improvements in muscle strength and functional reach [38]. Furthermore, gradually increasing the inclination angle and improving the gait speed provide repetitive interactions and high-intensity task-specific treatments for the hip, knee, and ankle joints. This approach is believed to enhance exercise recovery through neuroplasticity [39].

### 4.2. Functional Recovery

Balance is the primary focus of rehabilitation in patients with chronic stroke, as it is the most common cause of stroke. Balance is associated with increased postural sway, asymmetric weight distribution, decreased postural stability, and impaired weight-shifting ability. These issues can restrict functional activities and daily life functions [40]. Robot-assisted rehabilitation training enhances exercise learning and promotes functional recovery through task-specific repetitive approaches. It enhances the confidence of patients with stroke in their balance-related abilities. Additionally, it induces physiological muscle activation patterns through alternating movements and is effective in improving symmetrical posture [41].

In this study, balance significantly increased in the rehabilitation robot training group, as evidenced by a significant increase in the Berg Balance Scale (BBS) scores (*p* < 0.05). There was no significant difference between the rehabilitation robot training and control groups in between-group comparisons. However, there was a significant difference in the time × group interaction (*p* < 0.05). In terms of balance, the Functional Balance Grade (FBG) significantly increased within the rehabilitation robot training group in both the static and dynamic seated positions (*p* < 0.05), and there were significant differences between the groups in the time × group interaction effect for both the static and dynamic seated positions (*p* < 0.05). When examining previous studies on robot-assisted rehabilitation training and balance in stroke patients, Heng et al. [42] randomly assigned 24 patients with chronic stroke to two groups: a robot-assisted gait training group consisting of 12 patients and a traditional rehabilitation training group consisting of 12 patients. They evaluated the improvement in outcome variables between the groups based on balance measurements using the Berg Balance Scale (BBS) before and after training. The robot-assisted gait training group showed an increase in BBS scores from 26.73 points before training to 42.64 points after training, whereas the traditional rehabilitation training group increased from 32.18 points to 35.64 points. A significant difference was observed in the time × group interaction effect (*p* < 0.05). In the post-hoc analysis, a significant improvement was observed in the robot-assisted gait training group (*p* = 0.001), whereas there was no significant improvement in the traditional rehabilitation training group (*p* = 0.252). Kawamoto et al. [43] conducted robotic training sessions with 16 chronic stroke patients. The training was administered twice a week, with each session lasting 20–30 min, for a total of 16 sessions. They evaluated the improvement in outcome variables within each group based on balance measurements using the Berg Balance Scale (BBS) before and after training. The average change in BBS was 7.0 points, with scores increasing significantly from 40.6 points before training to 45.4 points after training (*p* < 0.05). The results of this study indicate, similar to the studies conducted by Heng et al. [42] and Kawamoto et al. [43], that robot-assisted rehabilitation training is an effective intervention method for improving balance in patients with subacute stroke, as evidenced by improvements in BBS scores. However, unlike the studies by Heng et al. [42] and Kawamoto et al. [43], this study differentiated between static balance and dynamic balance, as well as between seated and standing positions, using the Functional Balance Grade (FBG).

Between-group comparisons revealed significant differences in FBG levels in both the static and dynamic seated positions (*p* < 0.05). Additionally, there were significant differences in the time × group interaction effect for both static and dynamic seated-position FBG levels (*p* < 0.05). Therefore, robot-assisted rehabilitation training has demonstrated effectiveness as an intervention method for balance even in seated positions. From these results, it can be inferred that balance impairment in stroke patients stems from insufficient trunk muscle strength. Robot-assisted rehabilitation training likely positively affects balance stability by promoting alignment and postural symmetry [44]. Furthermore, in this study, the symmetrical vertical posture achieved during the robot-assisted rehabilitation training was presumed to encourage the balanced use of muscles on both sides and enhance proprioception, aiding in the improvement of balance sensation.

Robot-assisted rehabilitation training, particularly with the Erigo^®^Pro, involves lower limb stepping and verticalization. This intervention enhanced orthostatic tolerance in patients with stroke and consciousness disorders. Moreover, it improved the overall motor function and sensorimotor system plasticity, including proprioception and vestibular function. [23]. In this study, robot-assisted rehabilitation training involved gradual and steady verticalization of the patient while maintaining stability. Throughout this process, patients are required to perform various motor functions such as adjusting the upper and lower body, maintaining balance, and controlling posture. These exercises demand the stimulation of various areas of the brain and are presumed to promote neuroplasticity and recovery by facilitating brain readjustment and resilience.

### 4.3. ADL

Functional independence in activities of daily living is a crucial factor in predicting the prognosis of recovery in stroke patients. The loss of functional independence is commonly observed in patients with stroke. When patients with acute stroke receive rehabilitation therapy for six months, they often achieve the highest level of functional independence, which is significantly associated with an improved quality of life [45,46]. In this study, concerning functional independence in activities of daily living, there was a significant increase in FIM scores within the rehabilitation robot training group compared to the baseline. Similarly, there was a significant increase in the FIM scores in the control group (*p* < 0.05).

There were significant differences between the groups in the time × group interaction effect on functional independence in activities of daily living. (*p* < 0.05) Taveggia et al. [47] divided 28 acute stroke patients into a robot therapy group and a conventional walking therapy group through a double-blind randomized controlled trial. The participants underwent 25 treatment sessions over 5 weeks, 5 days a week.

They evaluated the improvement in outcome variables within each group and between groups using the Functional Independence Measure (FIM) for functional independence. The FIM was measured before and after treatment, as well as during the follow-up assessments for each group. In the robot therapy group, the Functional Independence Measure (FIM) scores increased significantly from 75.6 points before treatment to 89.4 points after treatment and further increased to 100.1 points during follow-up assessments. There was a significant improvement in the mean scores within the group. (*p* < 0.05). In the conventional walking therapy group, the Functional Independence Measure (FIM) scores increased from 90.8 points before treatment to 100.2 points after treatment and further increased to 100.6 points during follow-up assessments. However, there was no significant difference in the average scores between the groups. The interaction effect between time and group was not significant; however, there was a significant difference between groups (*p* < 0.003).

Robot-assisted therapy showed a significant improvement in FIM scores compared with conventional gait therapy. In this study, rehabilitation robot training demonstrated effectiveness as an intervention method for functional independence in subacute stroke patients, similar to the findings of Taveggia et al. [47]. In contrast to their study, the results of this study provide evidence of the effectiveness of robot-assisted therapy as an intervention method, as significant differences were found in both the intergroup comparisons and the time × group interaction effect.

In activities of daily living, functional limitations in participation occur due to motor and sensory impairments resulting from stroke [48].

Moreover, approximately half of stroke survivors depend on activities of daily living for their livelihoods. Physical therapy aimed at recovery and maintenance of activities of daily living is a core aspect of stroke rehabilitation [49]. In this study, there was a significant increase in the activities of daily living in the robot-assisted training group (*p* < 0.05). Significant differences were found in both intergroup comparisons and time × group interaction effects (*p* < 0.05). When examining previous research on rehabilitation robot training and activities of daily living in stroke patients, Kamdi et al. [50] divided 54 stroke patients into two groups: a robot therapy group receiving three sessions per day for four weeks and a conventional therapy group performing walking exercises five days a week for two weeks. They measured the MBI at admission and at two weeks and four weeks after admission. They evaluated improvements in outcome variables within and between groups using the MBI for activities of daily living. In the robot therapy group, the MBI increased significantly from 83.5 points at baseline to 90.0 points at two weeks and 93.5 points at four weeks (*p* < 0.001). 

Compared with the conventional therapy group, the MBI increased significantly from 87.8 points at baseline to 90.3 points at two weeks and 93.1 points at four weeks (*p* < 0.001). There was no significant difference found in intergroup comparisons; however, owing to the significant time × group interaction effect (*p* < 0.05), robotic therapy was more effective in improving MBI scores than conventional therapy. The results of this study indicate that rehabilitation robot training, similar to the findings of Kamdi et al. [50], is an effective intervention method for activities of daily living. In contrast to the study by Kamdi et al. [50], the results of this study demonstrated significant differences in both intergroup comparisons and the time × group interaction effect, thereby proving the effectiveness of the intervention method. 

Rehabilitation robot training has been reported to be effective in improving the daily living abilities of patients with stroke by providing repetitive and quantifiable therapy to help establish normal movement patterns [51]. 

In this study, rehabilitation robot training adjusted the parameters according to the patient’s motor abilities, allowing them to repeat movements tailored to their capabilities and maintain precise movement ranges and patterns. Through rehabilitation robot training, the movements necessary for performing activities of daily living can be enhanced by retraining the impaired motor function.

Based on the results of this study, robotic rehabilitation training for patients with subacute stroke is associated with lower limb strength in physical function, balance and motor function recovery in functional recovery, cognitive function in activities of daily living, and functional independence in daily activities. Therefore, in clinical settings, incorporating rehabilitation robot training alongside conventional physical therapy, occupational therapy, speech therapy, and rehabilitation equipment training tailored to each patient’s stage of physical recovery can lead to positive outcomes in subacute stroke patients.

This study has limitations in explaining the research findings. Firstly, the number of subjects is limited to 35, and it is difficult to generalize to the entire stroke population by targeting patients in Seoul and Gyeonggi Province in Korea. Secondly, it is challenging to generalize to the entire stroke population by targeting patients with bilateral stroke. Thirdly, although a progressive training protocol was used each week, the difficulty was adjusted based on the patient’s condition, so the same training intensity could not be provided. Fourthly, this study used a pre–post controlled group design to investigate the effects of 4 weeks of rehabilitation robot training on the physical function, functional recovery, and activities of daily living in subacute stroke patients, but long-term follow-up studies are needed. In future research, increasing the number of subjects; distinguishing between right, left, and bilateral stroke locations; and conducting further research on the effects of rehabilitation robot training on physical function, functional recovery, and activities of daily living in subacute stroke patients using the same protocol are deemed necessary.

## 5. Conclusions

This study aimed to investigate the effects of 4 weeks of rehabilitation robot training on physical function, functional recovery, and activities of daily living in patients with subacute stroke. Before and after training, changes in scores were measured using the MMT, BBS, FBG, FAC, FIM, and MBI to assess physical function, functional recovery, and activities of daily living. The following results were obtained: In terms of physical function, the rehabilitation robot training group showed statistically significant differences in flexion and extension muscle strengths of the hip, knee, and ankle joints across time, group, and time × group interaction effects. Thus, it can be concluded that rehabilitation robot training is effective in increasing lower limb strength. Second, in terms of functional recovery, the rehabilitation robot training group showed statistically significant differences in sitting balance ability, as measured by the static seated posture FBG and dynamic seated posture FBG across time, group, and time × group interaction effects. Therefore, it can be concluded that rehabilitation robot training is effective in increasing sitting balance. Third, in terms of daily living, the rehabilitation robot training group showed statistically significant differences in the FIM and MBI across time, group, and time × group interactions. Therefore, it can be concluded that rehabilitation robot training is effective in increasing the activities of daily living. This study confirmed that rehabilitation robot training is effective in improving physical function, functional recovery, and activities of daily living. The results of this study, along with those of various other treatment methods, will serve as foundational data for future research aimed at applying rehabilitation robot training to patients with subacute stroke in clinical settings.

## Figures and Tables

**Table 1:** Rehabilitation robot training with Erigo^®^ Pro.

Classification	Key Contents			Figure
Advantages	- The Erigo Pro gradually brings the patient into an upright position while moving the legs and applying cyclic leg loading. - This enables safe verticalization and early functional mobilization of the lower extremity, optionally supported by functional electrical stimulation. - Patients can be trained intensively and safely in a very early stage of rehabilitation.			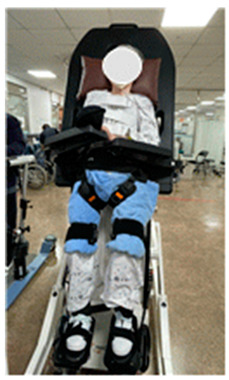 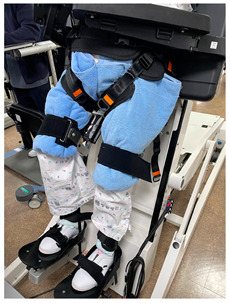
Key Features	- Sets appropriate training parameters for patients - Raises the inclination of the stepper and tilt table to induce upright posture - Enables upright posture while repetitive leg loading is applied to the lower limbs			
Preparation Process	- The subject wears shoes and lies flat on the inclined surface, aligning the hip joint area with the mat and harness lines and placing the feet on the footrest for alignment. - The shoulders, waist, pelvis, and ankles are secured with attached belts and straps. - The safety of the subject’s movement of the hip and knee joints is ensured by fitting the knee cups about three fingers (approximately 3–4 cm) above the kneecaps.			
Progressive Training Protocol	Weeks	Verticalization (tilt angle)	Cyclic leg loading (cadence)	
	1	20	40	
	2	40	45	
	3	60	45	
	4	80	50	
- Adjusts parameters based on the subject’s cardiovascular status, fatigue, concentration, adaptability, etc., to set difficulty levels - Can provide passive assistance with different values on each side depending on the subject’s condition - The ROM (range of motion) of each leg’s hip and knee joints is measured using sensors built into the training device, and the device’s software controls robotic leg movement within the ROM limits. - Conducted for 20 sessions over 4 weeks, 5 times a week, for 30 min each day				

**Table 2:** General characteristics of participants (*n* = 35).

Characteristics	RRT Group (*n* = 20)	Control Group (*n* = 15)	X^2^/*t(p)*
Gender (M/F)	13 (65.0) ^a^/7 (35.0)	10 (66.7)/5 (33.3)	0.100 (0.921)
Age (years)	63.40 (16.50) ^b^	60.40 (15.90)	0.541 (0.592)
Height (cm)	166.65 (10.30)	165.93 (9.58)	0.210 (0.835)
Weight (kg)	59.75 (10.69)	60.60 (21.26)	−0.155 (0.878)
Stroke type (I/H)	5 (25.0)/15 (75.0)	4 (26.7)/11 (73.3)	0.108 (0.914)
Lesion sites (bilateral)	20 (100.0)	15 (100.0)	1.000 (1.000)
Onset period (month)	3.65 (1.46)	3.47 (1.92)	0.321 (0.750)

^a^*n* (%); ^b^ M(SD); I = infarction; H = hemorrhage; RRT = rehabilitation robot training group.

**Table 3:** Comparison of physical function (*n* = 35).

Parameters		RRT Group (*n* = 20)	Control Group (*n* = 15)	Time F(p)	Group F(p)	Time × Group F(p)
Hip flexors (scores)	Before	2.40 (1.60) ^a^	1.33 (0.72)	38.110 (0.000)	18.461 (0.000)	33.758 (0.000)
	After	4.60 (2.19)	1.40 (0.91)			
	z(p) ^b^	−3.758 (0.000)	−1.000 (0.317)			
Hip extensors (scores)	Before	2.35 (1.60)	1.33 (0.72)	22.355 (0.000)	17.732 (0.000)	22.355 (0.000)
	After	4.45 (2.30)	1.33 (0.72)			
	z(p)	−3.555 (0.000)	0.000 (1.000)			
Knee flexors (scores)	Before	2.25 (1.51)	1.27 (0.59)	19.429 (0.000)	17.589 (0.000)	16.820 (0.000)
	After	4.10 (2.10)	1.33 (0.72)			
	z(p)	−3.345 (0.001)	−1.000 (0.317)			
Knee extensors (scores)	Before	2.25 (1.51)	1.40 (1.06)	13.984 (0.001)	11.515 (0.002)	11.952 (0.002)
	After	3.95 (2.19)	1.47 (1.13)			
	z(p)	−3.203 (0.001)	−1.000 (0.317)			
Dorsiflexors (scores)	Before	2.20 (1.70)	1.13 (0.35)	11.267 (0.002)	13.053 (0.001)	9.537 (0.004)
	After	3.80 (2.50)	1.20 (0.56)			
	z(p)	−3.022 (0.003)	−1.000 (0.317)			
Plantar flexors (scores)	Before	2.25 (1.74)	1.27 (0.59)	12.411 (0.001)	13.149 (0.001)	10.608 (0.003)
	After	3.95 (2.35)	1.33 (0.72)			
	z(p)	−3.031 (0.002)	−1.000 (0.317)			

^a^ M(SD); ^b^ Within-group pairwise comparisons were conducted using the Wilcoxon signed-rank test; MMT = manual muscle test; RRT = rehabilitation robot training group.

**Table 4:** Comparison of functional recovery (*n* = 35).

Parameters		RRT Group (*n* = 20)	Control Group (*n* = 15)	Time F(p)	Group F(p)	Time × Group F(p)
BBS (scores)	Before	1.60 (4.88) ^a^	0.26 (1.03)	6.100 (0.019)	3.193 (0.083)	4.236 (0.048)
	After	6.00 (10.23)	0.67 (1.80)			
	t(p)	−2.646 (0.016)	−1.382 (0.189)			
SSeP FBG (scores)	Before	0.65 (1.04)	0.20 (0.77)	16.456 (0.000)	6.525 (0.015)	6.306 (0.017)
	After	1.50 (1.10)	0.40 (0.83)			
	z(p) ^b^	−3.002 (0.003)	−1.732(0.083)			
DSeP FBG (scores)	Before	0.35 (0.88)	0.13 (0.52)	8.214 (0.007)	5.030 (0.046)	8.214 (0.007)
	After	1.00 (1.12)	0.13 (0.52)			
	z(p)	−2.754 (0.006)	0.000 (1.000)			
SStP FBG (scores)	Before	0.15 (0.49)	0.00 (0.00)	3.536 (0.069)	3.628 (0.066)	3.536 (0.069)
	After	0.55 (1.05)	0.00 (0.00)			
	z(p)	−2.060 (0.039)	0.000 (1.000)			
DStP FBG (scores)	Before	0.10 (0.31)	0.00 (0.00)	3.075 (0.089)	3.023 (0.091)	3.075 (0.089)
	After	0.35 (0.75)	0.00 (0.00)			
	z(p)	−1.890 (0.059)	0.000 (1.000)			
FAC (scores)	Before	0.50 (0.22)	0.00 (0.00)	4.228 (0.048)	2.899 (0.098)	2.706 (0.109)
	After	0.65 (1.31)	0.07 (0.26)			
	z(p)	−2.232 (0.026)	−1.000 (0.317)			

^a^ M(SD); ^b^ Within-group pairwise comparisons were conducted using the Wilcoxon signed-rank test; RRT = rehabilitation robot training group; BBS = Berg’s Balance Scale; SseP = static seated posture; DseP = dynamic seated posture; SStP = static standing posture; DStP = dynamic standing posture; FBG = Functional Balance Grade; FAC = Functional Ambulation Category.

**Table 5:** Comparison of ADL (*n* = 35).

Parameters		RRT Group (*n* = 20)	Control Group (*n* = 15)	Time F(p)	Group F(p)	Time × Group F(p)
FIM (scores)	Before	23.25 (6.78) ^a^	20.07 (3.35)	10.129 (0.003)	5.490 (0.025)	4.916 (0.034)
	After	36.30 (21.86)	22.40 (7.12)			
	t(p)	−3.185 (0.005)	−2.233 (0.042)			
MBI (scores)	Before	4.15 (8.34)	0.73 (1.87)	7.557 (0.010)	4.550 (0.040)	4.285 (0.046)
	After	16.45 (24.36)	2.47 (5.67)			
	t(p)	−2.837 (0.011)	−1.660 (0.119)			

^a^ M(SD); RRT = rehabilitation robot training group; FIM = Functional Independence Measure; MBI = Modified Barthel Index.

## Data Availability

Data are contained within the article.

## References

[1] Dionísio A., Duarte I.C., Patrício M., Castelo-Branco M. (2018). The Use of Repetitive Transcranial Magnetic Stimulation for Stroke Rehabilitation: A Systematic Review. J. Stroke Cerebrovasc. Dis..

[2] (2021). Korean Statistical Information Service. https://kosis.kr/index/index.do.

[3] Warutkar V., Dadgal R., Mangulkar U.R. (2022). Use of Robotics in Gait Rehabilitation Following Stroke: A Review. Cureus.

[4] Miss B.M., Gund G. (2013). Stroke: A Brain Attack. IOSR J. Pharm..

[5] Al-Qazzaz N.K., Ali S.H., Ahmad S.A., Islam S., Mohamad K. (2014). Cognitive impairment and memory dysfunction after a stroke diagnosis: A post-stroke memory assessment. Neuropsychiatr. Dis. Treat..

[6] Saunders D.H., Sanderson M., Hayes S., Kilrane M., Greig C.A., Brazzelli M., Mead G.E. (2016). Physical fitness training for stroke patients. Cochrane Database Syst. Rev..

[7] Kazi F., Dadgal R., Salphale V.G. (2022). Impact of Proprioceptive Neuromuscular Facilitation Technique for Early Rehabilitation to Restore Motor Impairments in a Classic Case of Left Middle Cerebral Artery Stroke. Cureus.

[8] Penna L.G., Pinheiro J.P., Ramalho S.H.R., Ribeiro C.F. (2021). Effects of aerobic physical exercise on neuroplasticity after stroke: Systematic review. Arq. Neuropsiquiatr..

[9] Prigatano G.P., Braga L.W., Johnson S.F., Souza L.M.N. (2021). Neuropsychological rehabilitation, neuroimaging and neuroplasticity: A clinical commentary. NeuroRehabilitation.

[10] Lin S.H., Dionne T.P. (2018). Interventions to Improve Movement and Functional Outcomes in Adult Stroke Rehabilitation: Review and Evidence Summary. J. Particip. Med..

[11] Inoue S., Otaka Y., Kumagai M., Sugasawa M., Mori N., Kondo K. (2022). Effects of Balance Exercise Assist Robot training for patients with hemiparetic stroke: A randomized controlled trial. J. Neuroeng. Rehabil..

[12] Mijic M., Schoser B., Young P. (2023). Efficacy of functional electrical stimulation in rehabilitating patients with foot drop symptoms after stroke and its correlation with somatosensory evoked potentials-a crossover randomised controlled trial. Neurol. Sci..

[13] Mahmood W., Ahmed Burq H.S.I., Ehsan S., Sagheer B., Mahmood T. (2022). Effect of core stabilization exercises in addition to conventional therapy in improving trunk mobility, function, ambulation and quality of life in stroke patients: A randomized controlled trial. BMC Sports Sci. Med. Rehabil..

[14] Hu Y., Tian J., Wen X., Lu C., Tian N. (2022). Clinical Effects of MOTOmed Intelligent Exercise Training Combined with Intensive Walking Training on the Rehabilitation of Walking, Nerve and Lower Limb Functions among Patients with Hemiplegia after Stroke. Pak. J. Med. Sci..

[15] van de Port I.G., Wevers L.E., Lindeman E., Kwakkel G. (2012). Effects of circuit training as alternative to usual physiotherapy after stroke: Randomised controlled trial. BMJ.

[16] Liu Y.C., Yang Y.R., Tsai Y.A., Wang R.Y. (2017). Cognitive and motor dual task gait training improve dual task gait performance after stroke—A randomized controlled pilot trial. Sci. Rep..

[17] Linder S.M., Rosenfeldt A.B., Davidson S., Zimmerman N., Penko A., Lee J., Clark C., Alberts J.L. (2019). Forced, Not Voluntary, Aerobic Exercise Enhances Motor Recovery in Persons with Chronic Stroke. Neurorehabil. Neural Repair..

[18] Calabrò R.S., Naro A., Russo M., Leo A., Balletta T., Saccá I., De Luca R., Bramanti P. (2015). Do post-stroke patients benefit from robotic verticalization? A pilot-study focusing on a novel neurophysiological approach. Restor. Neurol. Neurosci..

[19] Rowe J.B., Chan V., Ingemanson M.L., Cramer S.C., Wolbrecht E.T., Reinkensmeyer D.J. (2017). Robotic Assistance for Training Finger Movement Using a Hebbian Model: A Randomized Controlled Trial. Neurorehabil. Neural Repair.

[20] Turolla A., Kiper P., Mazzarotto D., Cecchi F., Colucci M., D’Avenio G., Molteni F. (2022). Reference theories and future perspectives on robot-assisted rehabilitation in people with neurological conditions: A scoping review and recommendations from the Italian Consensus Conference on Robotics in Neurorehabilitation (CICERONE). NeuroRehabilitation.

[21] Masiero S., Poli P., Rosati G., Zanotto D., Iosa M., Paolucci S., Morone G. (2014). The value of robotic systems in stroke rehabilitation. Expert Rev. Med. Devices.

[22] Takeuchi N., Izumi S.-I. (2013). Rehabilitation with Poststroke Motor Recovery: A Review with a Focus on Neural Plasticity. Stroke Res. Treat..

[23] Rosenfelder M.J., Helmschrott V.C., Willacker L., Einhäupl B., Raiser T.M., Bender A. (2023). Effect of robotic tilt table verticalization on recovery in patients with disorders of consciousness: A randomized controlled trial. J. Neurol..

[24] De Luca R., Bonanno M., Vermiglio G., Trombetta G., Andidero E., Caminiti A., Pollicino P., Rifici C., Calabrò R.S. (2022). Robotic Verticalization plus Music Therapy in Chronic Disorders of Consciousness: Promising Results from a Pilot Study. Brain Sci..

[25] Ueda K., Umemoto Y., Kamijo Y.-i., Sakurai Y., Araki S., Ise M., Yoshioka I., Banno M., Mochida S., Iwahashi T. (2022). Effects of Combination of Functional Electric Stimulation and Robotic Leg Movement Using Dynamic Tilt Table on Walking Characteristics in Post-Stroke Patients with Spastic Hemiplegia: A Randomized Crossover-Controlled Trial. J. Clin. Med..

[26] Pathak A., Gyanpuri V., Dev P., Dhiman N.R. (2021). The Bobath Concept (NDT) as rehabilitation in stroke patients: A systematic review. J. Family Med. Prim. Care.

[27] Mikołajewska E. (2012). NDT-Bobath method in normalization of muscle tone in post-stroke patients. Adv. Clin. Exp. Med..

[28] Krukowska J., Bugajski M., Sienkiewicz M., Czernicki J. (2016). The influence of NDT-Bobath and PNF methods on the field support and total path length measure foot pressure (COP) in patients after stroke. Neurol. Neurochir. Pol..

[29] Baschung Pfister P., de Bruin E.D., Sterkele I., Maurer B., de Bie R.A., Knols R.H. (2018). Manual muscle testing and hand-held dynamometry in people with inflammatory myopathy: An intra- and interrater reliability and validity study. PLoS ONE.

[30] Blum L., Korner-Bitensky N. (2008). Usefulness of the Berg Balance Scale in stroke rehabilitation: A systematic review. Phys. Ther..

[31] Butsara C., Suthisa P., Somchanok H., Rumpa B. (2020). Reliability of the Modified O’Sullivan Functional Balance Test in Person with Spinal Cord Injury. J. Food Health Bioenviron. Sci..

[32] Mehrholz J., Wagner K., Rutte K., Meissner D., Pohl M. (2007). Predictive validity and responsiveness of the functional ambulation category in hemiparetic patients after stroke. Arch. Phys. Med. Rehabil..

[33] Granger C.V., Cotter A.C., Hamilton B.B., Fiedler R.C. (1993). Functional assessment scales: A study of persons after stroke. Arch. Phys. Med. Rehabil..

[34] Ohura T., Hase K., Nakajima Y., Nakayama T. (2017). Validity and reliability of a performance evaluation tool based on the modified Barthel Index for stroke patients. BMC Med. Res. Methodol..

[35] Shao C., Wang Y., Gou H., Xiao H., Chen T. (2023). Strength Training of the Nonhemiplegic Side Promotes Motor Function Recovery in Patients with Stroke: A Randomized Controlled Trial. Arch. Phys. Med. Rehabil..

[36] Molteni F., Gasperini G., Cannaviello G., Guanziroli E. (2018). Exoskeleton and End-Effector Robots for Upper and Lower Limbs Rehabilitation: Narrative Review. PM R.

[37] Kumar S., Yadav R., Aafreen (2020). Comparison between Erigo tilt-table exercise and conventional physiotherapy exercises in acute stroke patients: A randomized trial. Arch. Physiother..

[38] Kuznetsov A.N., Rybalko N.V., Daminov V.D., Luft A.R. (2013). Early poststroke rehabilitation using a robotic tilt-table stepper and functional electrical stimulation. Stroke Res. Treat..

[39] Geiger D.E., Behrendt F., Schuster-Amft C. (2019). EMG Muscle Activation Pattern of Four Lower Extremity Muscles during Stair Climbing, Motor Imagery, and Robot-Assisted Stepping: A Cross-Sectional Study in Healthy Individuals. Biomed. Res. Int..

[40] Alghadir A.H., Al-Eisa E.S., Anwer S., Sarkar B. (2018). Reliability, validity, and responsiveness of three scales for measuring balance in patients with chronic stroke. BMC Neurol..

[41] Lin Y.N., Huang S.W., Kuan Y.C., Chen H.C., Jian W.S., Lin L.F. (2022). Hybrid robot-assisted gait training for motor function in subacute stroke: A single-blind randomized controlled trial. J. Neuroeng. Rehabil..

[42] Heng H.M., Lu M.K., Chou L.W., Meng N.H., Huang H.C., Hamada M., Tsai C.H., Chen J.C. (2020). Changes in Balance, Gait and Electroencephalography Oscillations after Robot-Assisted Gait Training: An Exploratory Study in People with Chronic Stroke. Brain Sci..

[43] Kawamoto H., Kamibayashi K., Nakata Y., Yamawaki K., Ariyasu R., Sankai Y., Sakane M., Eguchi K., Ochiai N. (2013). Pilot study of locomotion improvement using hybrid assistive limb in chronic stroke patients. BMC Neurol..

[44] De Luca A., Squeri V., Barone L.M., Vernetti Mansin H., Ricci S., Pisu I., Cassiano C., Capra C., Lentino C., De Michieli L. (2020). Dynamic Stability and Trunk Control Improvements Following Robotic Balance and Core Stability Training in Chronic Stroke Survivors: A Pilot Study. Front. Neurol..

[45] González-Santos J., Rodríguez-Fernández P., Pardo-Hernández R., González-Bernal J.J., Fernández-Solana J., Santamaría-Peláez M. (2023). A Cross-Sectional Study: Determining Factors of Functional Independence and Quality of Life of Patients One Month after Having Suffered a Stroke. Int. J. Environ. Res. Public Health.

[46] Bisevac E., Lazovic M., Nikolic D., Mahmutovic E., Dolicanin Z., Jurisic-Skevin A. (2022). Postacute Rehabilitation Impact on Functional Recovery Outcome and Quality of Life in Stroke Survivors: Six Month Follow-Up. Medicina.

[47] Taveggia G., Borboni A., Mulé C., Villafañe J.H., Negrini S. (2016). Conflicting results of robot-assisted versus usual gait training during postacute rehabilitation of stroke patients: A randomized clinical trial. Int. J. Rehabil. Res..

[48] Guiu-Tula F.X., Cabanas-Valdés R., Sitjà-Rabert M., Urrútia G., Gómara-Toldrà N. (2017). The Efficacy of the proprioceptive neuromuscular facilitation (PNF) approach in stroke rehabilitation to improve basic activities of daily living and quality of life: A systematic review and meta-analysis protocol. BMJ Open.

[49] García-Rudolph A., Sánchez-Pinsach D., Salleras E.O., Tormos J.M. (2019). Subacute stroke physical rehabilitation evidence in activities of daily living outcomes: A systematic review of meta-analyses of randomized controlled trials. Medicine.

[50] Kamdi M.K.A., Shafei M.N., Musa K.I., Hanafi M.H., Suliman M.A. (2023). Comparison of the Modified Barthel Index (MBI) Score Trends Among Workers with Stroke Receiving Robotic and Conventional Rehabilitation Therapy. Cureus.

[51] Qu Q., Lin Y., He Z., Fu J., Zou F., Jiang Z., Guo F., Jia J. (2021). The Effect of Applying Robot-Assisted Task-Oriented Training Using Human-Robot Collaborative Interaction Force Control Technology on Upper Limb Function in Stroke Patients: Preliminary Findings. Biomed. Res. Int..

